# Medical adhesive-related skin injury in adult intensive care unit: scoping review

**DOI:** 10.1590/0034-7167-2021-0926

**Published:** 2022-09-09

**Authors:** Ariana Luiza Rabelo, Jéssica Bordonal, Thays Lopes de Almeida, Patrícia Peres Oliveira, Juliano Teixeira Moraes

**Affiliations:** I Universidade Federal de São João Del Rei. Divinópolis, Minas Gerais, Brazil

**Keywords:** Skin, Wounds and Injuries, Intensive Care Units, Critical Care, Enterostomal Therapy, Piel, Heridas y Lesiones, Unidades de Cuidados Intensivos, Cuidados Críticos, Estomaterapia, Pele, Ferimentos e Lesões, Unidades de Terapia Intensiva, Cuidados Críticos, Estomaterapia

## Abstract

**Objectives:**

to identify and synthesize scientific evidence on preventing medical adhesive-related skin injuries in adult intensive care patients.

**Methods:**

this is a scoping review based on PRISMA-ScR recommendations and the technique proposed by Joanna Briggs Institute. PubMed, CINAHL, Web of Science, Scopus, LILACS, and Embase databases were searched using “Injuries AND Adhesives AND Skin AND Medical” descriptors”.

**Results:**

1,329 studies were identified, and after analysis, the final sample consisted of nine articles. We obtained two experts’ consensus, three case studies, two cross-sectional studies, one prospective cohort study, and one literature review regarding the type of studies.

**Final Considerations:**

the synthesized evidence allowed us to list health care measures to prevent medical adhesive-related skin injuries. The professional must know how to identify the skin injuries associated with medical adhesives and the main strategies for their prevention.

## RESUMO

**Objetivos:**

identificar e sintetizar evidências científicas sobre prevenção de lesões de pele relacionadas a adesivo médico nos pacientes adultos em terapia intensiva.

**Métodos:**

trata-se de uma *scoping review* desenvolvida com base nas recomendações do PRISMA-ScR e no método proposto por *Joanna Briggs Institute*. Realizaram-se buscas nas bases de dados PubMed, CINAHL, *Web of Science*, Scopus, LILACS e Embase, utilizando os descritores “*Injuries* AND *Adhesives* AND *Skin* AND *Medical*”.

**Resultados:**

identificaram-se 1.329 estudos; após análise, a amostra final foi composta por nove artigos. Quanto ao tipo de estudos, obtiveram-se dois consensos de especialistas, três estudos de caso, dois estudos transversais, um de coorte prospectivo e uma revisão de literatura.

**Considerações Finais:**

as evidências sintetizadas possibilitaram elencar cuidados para prevenção de lesões de pele relacionadas a adesivos médicos. É importante que o profissional saiba identificar tanto as lesões de pele relacionadas aos adesivos médicos quanto as principais estratégias para prevenção destas.

## RESUMEN

**Objetivos:**

identificar y sintetizar evidencias científicas sobre prevención de lesiones cutáneas relacionadas con adhesivo médico en pacientes adultos en cuidado intensivo.

**Métodos:**

se trata de una *scoping review* desarrollada basada en las recomendaciones del PRISMA-ScR y en el método propuesto por *Joanna Briggs Institute*. Realizadas búsquedas en las bases de datos PubMed, CINAHL, *Web of Science*, Scopus, LILACS y Embase, utilizando los descriptores “*Injuries* AND *Adhesives* AND *Skin* AND *Medical*”.

**Resultados:**

identificados 1.329 estudios; posterior análisis, el muestreo final fue compuesto por nueve artículos. Cuanto al tipo de estudios, obtenidos dos consensos de especialistas, tres estudios de caso, dos estudios transversales, uno de cohorte prospectivo y una revisión de literatura.

**Consideraciones Finales:**

las evidencias sintetizadas posibilitaron enumerar cuidados para prevención de lesiones cutáneas relacionadas con adhesivos médicos. Es importante que el profesional sepa identificar tanto las lesiones cutáneas relacionadas con adhesivos médicos cuanto las principales estrategias para prevención de estas.

## INTRODUCTION

Adhesive products are present in many different health care settings and services. In the intensive care unit (ICU), patients use many adhesive devices: monitoring electrodes, catheter fixators, vascular device attachments, endotracheal tube attachments, drains, and dressings. Therefore, critically ill patients are more exposed to medical adhesives and, consequently, an increased risk of adhesive-related skin injuries^(1-2)^.

We point out that “medical adhesive-related skin injuries” (MARSI) is a definition of any injury characterized by altered skin integrity, including erythema, laceration, erosion, blister, blebs, and others, that persists for 30 minutes or more after removal of an adhesive product^(2)^.

The differential diagnosis of MARSI in critically ill patients becomes challenging because some skin injuries may present similar characteristics, called “confounding lesions.” Identifying the etiology of the injury is critical for assertive diagnosis and nursing care management^(3)^. A prospective cohort study included incontinence-associated dermatitis, FI, and MARSI as confounding lesions for pressure injury. The estimated incidence was 35.6% in the intensive care unit setting^(4)^.

Injuries with common characteristics to MARSI include friction injuries (FI) and moisture-associated dermatitis (IAD). FIs are traumatic wounds that may result from various mechanical forces such as shear or friction forces, including blunt trauma, falls, equipment injury, or adhering dressings removal^(5)^. Particularly, IAD is a type of moisture-associated dermatitis defined as prolonged skin exposure to effluents, which may be urine, feces, exudate, or sweat, with erythema and edema of the surface, and accompanied by erosion, blisters with exudate, or secondary infection^(6)^.

In Brazil, epidemiological data on MARSI in adult intensive care unit settings are still scarce. A study conducted in a cardiology ICU showed a prevalence of 22.7%^(7)^. Worldwide, indicators differ depending on the geographic location. In Beijing, the injuries incidence in two ICUs was 10.96%^(1)^; still, in China, a cross-sectional study in four hospitals showed a prevalence of 19.7% in patients with the peripherally inserted central catheter (PICC)^(8)^; in the United States, the mean prevalence was 13% in two intensive care units^(9)^.

MARSI is an adverse event (AE) still not fully recognized by nursing and other health professionals. It is noteworthy that an AE can occur in any setting providing health care; however, its recognition can serve as a basis for decision-making and patient safety planning through analysis, monitoring, minimization, and prevention^(10)^.

Besides negatively impacting patient care and safety, medical adhesive-related skin injuries can influence the length of hospital stay, increase the period of treatment and its respective costs, and promote risks of infections and complications. Therefore, professionals should be aware of practices for managing medical adhesives and take care of prevention, diagnosis, and treatment^(8,11)^.

In addition to patients and professionals involved in care, the subject deserves attention from health services managers, who should consider its association with ethical, legal, and financial aspects. In that way, it is essential to explore scientific publications to expand the knowledge about medical adhesive-related skin injuries and, subsequently, about adherence to protocols, aiming at the quality of services provided.

## OBJECTIVES

To identify and summarize scientific evidence on preventing medical adhesive-related skin injuries in adult intensive care unit patients.

## METHODS

That is a scoping review, a systematic approach to map evidence and identify the main concepts, theories, sources, and gaps in knowledge on a given subject^(12-13)^. The research protocol was structured according to the recommendations of the international guide PRISMA-ScR^(12)^ and based on the technique proposed by Joanna Briggs Institute (JBI), which suggests five steps: 1) identification of the research question; 2) identification of relevant studies; 3) selection of studies; 4) data analysis; and 5) grouping, synthesis, and presentation of data^(13)^. That was registered in the Open Science Framework, registration N^o^ DOI 10.17605/OSF.IO/6QKAV.

We used the PCC strategy, P (Population) - adult patients; C (Concept) - medical adhesive-related skin injuries; and C (Context) - intensive care unit to formulate the guiding question, to develop the following research question: What scientific evidence is available in the context of adult intensive care unit to prevent medical adhesive-related skin injury?

The study conducted a preliminary search to identify similar scoping reviews and the primary descriptors and keywords used in studies that addressed the topic of interest. After that analysis, the final search strategy was defined as “Injuries AND Adhesives AND Skin AND Medical” and was carried out in May, June, and July 2021 in the PubMed, CINAHL, Web of Science, Scopus, LILACS, and Embase databases.

The inclusion criteria for the studies’ selection were scientific research which published the complete text, in English, Portuguese and Spanish, describing medical adhesive-related skin injuries and their prevention in adult intensive care unit patients. Exclusion criteria were publications in the format of articles, reviews, and letters. At the end of the search, we sent the studies to Rayyan^®^, a free application available on the internet, used to assist in research such as review and meta-analysis type, which allows streamlining the initial screening of abstracts and titles.

Three researchers did the initial evaluation of the studies. Initially, the title and abstract of all identified studies were analyzed. Based on the established criteria, the selected studies were read in their entirety to identify health care recommendations for preventing skin injuries related to medical adhesives. The data extracted from the studies were pre-established in the search protocol. A structured instrument identified the literature, with information about the title, study type, year of publication, country of origin, objective, type of research, population, location, description of care for prevention of MARSI, and results.

Those data, plus the classification of the level of evidence (LE), were summarized, grouped, and presented in Chart 1. The classification of the studies’ LE was implemented using the classification system recommended by the JBI, as follows: Level 5 (expert opinion), Level 4 (descriptive observational studies), Level 3 (analytical observational studies), Level 2 (quasi-experimental studies), and Level 1 (experimental studies)^(14)^.

**Chart 1:** Studies summarized according to title, authors, year, country, type of study, level of evidence and main preventive care

Title		Author/year	Country	LE^^*^^	Type of study	Objective	Preventive Care
A1	Medical adhesives and patient safety: state of the science consensus statements for the assessment prevention^(2)^	McNichol L, Lund C, Rosen T, Gray M. 2013	USA	5	Expert consensus	Establish consensus statements on the assessment, prevention, and treatment of MARSI.	Identification of high-risk patients. Skin care. Maintain good nutrition and hydration, select the most appropriate adhesive product, and assess skin conditions. Consider using a barrier film to the skin before applying an adhesive product. Limit the use of products that increase adherence to the adhesive. Use proper techniques for adhesive removal. Consider using removers.
A2	Minimising pain and medical adhesive- related skin injuries in vulnerable patients^(15)^	Collier, M. 2019	United Kingdom	4	Case Study	Review the literature about medical adhesive-related injuries and present an adhesive remover pointing out its benefits.	Recommends removal of adhesive products using Appeel Sterile, a sterile silicone medical adhesive remover clinically indicated for several patients whose skin is at risk of being easily compromised. It can be used on intact and injured skin around the central lines and for the safe removal of a variety of other medical devices and connections.
A3	Overlooked and underestimated: medical adhesive-related skin injuries. J Wound Care^(11)^	Fumarola S, Allaway R, Callaghan R, Collier M, Downie F, Geraghty J 2020	United Kingdom	5	Expert consensus	Increase awareness of MARSI. Encourage a change in culture whereby risk assessment and prevention of these injuries are considered essential parts of patient care.	All patients should have their skin routinely evaluated. The choice of medical adhesive should be based on the patient’s skin type, considering the risk factors. Perform proper technique for patch application and removal. Keep a skin care routine. Consider the use of barrier products and removers. Teach health professionals and patients about the subject and the preventive measures.
A4	Medical adhesive-related skin injuries associated with vascular access^(16)^	Hitchcock J, Savine L. 2017	United Kingdom	4	Case Study	Discuss the maintenance of skin integrity, association of products and dressings more appropriate and recognize patients at risk of MARSI, and protection of venous access.	Presents an intervention algorithm for preventing medical adhesive-related skin injuries. Recommendations: Remove the dressing slowly from the distal edges at a low angle, providing support to the skin at the removal line. Consider using a removal product. Apply a barrier film. Apply the dressing without tension, without pulling or stretching when the skin is dry. Address malnutrition and dehydration. Assess the skin daily. Record the procedure thoroughly.
A5	Prevalence and associated factors of medical adhesive-related skin injury in cardiac critical care units^(7)^	Alcântara CMP, Oliveira ELS, Campanili TCGF, Santos RSCS, Santos VLCG, Nogueira PC. 2021	Brazil	4	Cross-sectional study	Identify and analyze the point prevalence of MARSI in patients admitted to a cardiology intensive care unit and the demographic and clinical factors associated with its occurrence.	Appropriate choice of medical adhesive considering clinical purpose, anatomical location, adhesive properties, time of permanence, and skin conditions. Trim hair at the application site. When applying the liquid solution, allow it to dry completely to avoid humidity; avoid tension and asymmetrical areas at the time of application. During removal, take it off slowly, in a horizontal position, according to the hair growth, and hold the skin close to the removal point. Consider using an adhesive remover. Avoid excessive, unnecessary dressing changes.
A6	Clinical evaluation of a silicone adhesive remover for prevention of MARSI at dressing change^(17)^	Hadfield G, De Freitas A, Bradbury S 2021	United Kingdom	4	Case Study	Provide an overview of MARSI and its impact on clinical practice, followed by the results of a clinical evaluation of the effectiveness of a remover in preventing MARSI and dressing-related pain in vascular access devices.	A structured approach to assess risk and select the appropriate adhesive products. Consider proper techniques for applying and removing them. Normal skin and patient assessment. Train the staff on early recognition of MARSI. The use of silicone remover prevented further MARSI episodes, reduced existing skin damage, and decreased patient pain and anxiety during the dressing change procedure.
A7	Incidence and Influencing Factors of Medical Adhesive-Related Skin Injury in Critically Ill Patients^(1)^	Zhang YMS, Wang SBS, Zhang XMS, Zhang WBS, Wang XBS. 2020	China	3	Prospective cohort	Determine the incidence and factors influencing medical adhesive-related skin injury (MARSI) in ICU patients.	Follow good practice guidelines when applying and removing adhesive products to prevent MARSI. Do not use adhesive products with tension because this can cause stress injuries. It is recommended to smooth the adhesive product in its place with gentle, firm pressure, avoiding gaps and wrinkles. Removal of the adhesive should be slow while keeping the adhesive product horizontal and folded over on itself, with the skin support. An edge can also be folded, forming a (skin) flap to facilitate removal.
A8	Incidence of and Risk Factors for Medical Adhesive-Related Skin Injuries Among Patients: A Cross-sectional Study^(18)^	Gao C, Yu C, Lin X, Wang H, Sheng Y 2020	China	4	Cross-sectional study	Identify the incidence of MARSI injuries developed in an intensive care unit and to pinpoint relevant risk factors associated with the injuries.	It is necessary to follow good clinical practice recommendations to ensure patient safety. The occurrence of MARSI can be reduced by the proper use of appropriate adhesives, with more education and awareness. The suggested correct angle for removal of an adhesive is 0° or 180° in a very slow movement.
A9	Management of Central Venous Access Device-Associated Skin Impairment^(19)^	Broadhurst D, Moureau N, Ullman AJ 2017	Europe, Australia, and North American countries	4	Literature review	Develop an algorithm to improve the identification and diagnosis of skin injuries around central venous access; guide decision-making for the best management for that access; and improve the clinic confidence when caring for patients with skin injuries at the location of the central venous access device.	Interventions are described in three sequential domains: assessment, skin protection, and comfort. Training is a key strategy to prevent and manage skin compromise associated with central venous access. Many injuries can be prevented with the use of proper dressing application and removal techniques and application of antiseptics. Nurses and patients who perform dressing changes should be trained on how to safely perform the procedures to prevent injuries.

Note:

* *LE- level of evidence.*

## RESULTS

**Figure 1:**
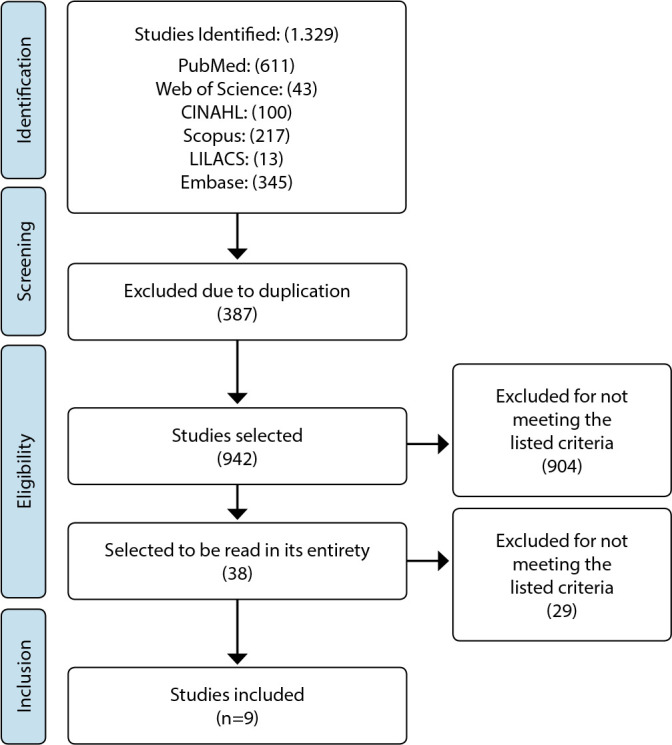
PRISMA Flowchart of the Study Selection Process, 2021

The initial database search generated 1.329 studies: 611 identified in PubMed, 43 in Web of Science, 100 in CINAHL, 217 in Scopus, 13 in LILACS, and 345 in Embase. Three hundred eighty-seven studies were excluded due to duplication in the initial screening process, leaving 942 articles for the title and abstract review. After analysis, we selected 38 articles to be read in their entirety. In the end, the sample was composed of nine articles. We detailed that process in the flowchart of Figure 1.

The articles selected were published between 2013 and 2021. The studies originated from the United States of America (1; 11.1%), United Kingdom (4; 44.4%), China (2; 22.2%), Brazil (1; 11.1%); and 1 (11.1%) was multicenter, including Europe, Australia, and North American countries. Overall, the studies did not have a strong level of evidence: two studies had Level 5 evidence (expert consensus), six studies presented Level 4 (3 case studies, two cross-sectional studies, and one literature review), and 1 study had Level 3 (prospective cohort).

We can describe the primary strategies for MARSI prevention in nursing care through the identified studies. Some medical care repeats itself throughout the articles, so it was possible to group them into categories presented in Chart 2.

**Chart 2:** Precautions to prevent medical adhesive-related skin injuries

Precautions to prevent medical adhesive-related skin injuries 1. Skin Assessment 2. Identification of patients at risk 3. Selection of product 4. Skin Preparation 5. Adhesive Application Technique 6. Adhesive Removal Technique 7. Continuing training of health professionals

## DISCUSSION

As observed, the studies identified do not present high levels of evidence. We want to point out that the discussion on this subject is new. The first international consensus on MARSI was published in 2013^(2)^, raising interest in further studies. The concept on the subject is not yet extensively disseminated among nursing professionals. Therefore, it is essential to emphasize the need for more clinical studies to support evidence-based practice.

Nevertheless, the publications mentioned address strategies that consider the approach to MARSI prevention care, such as skin assessment, identification of patients at risk, selection of product, skin preparation, adhesive application and removal techniques, and the continuing training of health professionals.

Assessing the skin is a particularly relevant nursing intervention, mainly in patients exposed to medical adhesives. Studies report that the patients’ skin should be evaluated in the admission process, at regular intervals, before and after using adhesive products. During that process, the professional should pay attention to these characteristics: skin integrity, color, temperature, fragility, edema, and signs of local irritation^(2,11,17)^.

But the detection of risk factors will help professionals identify patients at risk of developing skin injuries. The literature mentions those factors, such as extremes of age, underlying dermatological conditions, some comorbidities (diabetes, renal failure, hypertension, venous insufficiency, among others), skin dryness, prolonged use of corticosteroids, coagulants, dehydration, malnutrition, exposure to moisture, use of adhesive tapes and products^(1-2,7,17)^.

The choice of the adhesive product is essential. For that, besides the information collected during skin assessment and the identification of risk factors, it is necessary to consider aspects such as anatomical position, region of movement, exposure or not to moisture, presence of edema, estimated time of adhesive permanence, purpose, and characteristics of each adhesive product^(9,11,18)^. Before applying the patch, the skin has to be clean and dry, and the hair trimmed if necessary. It should employ skin barrier products because they provide a protective interface between the adhesive and the skin. Furthermore, avoid substances that boost the strength of the patch of the medical adhesive^(2,11,16,18)^.

Proper application and removal of the adhesive product are paramount to minimize skin damage. The adhesive must be applied without tension and placed with firm and gentle compression, avoiding gaps and folds. During removal, patches can damage the skin and cause pain to the patient, so removal products should be considered because, besides removing adhesive residues and the barrier film, they minimize discomfort and damage to the skin. Furthermore, the appropriate technique recommended for removal is to start it from the edges, slowly, at a low angle, in the direction of hair growth, keeping it horizontally, close to the skin surface^(1-2,11,18)^.

Training of health professionals is a crucial strategy for the processes described above. Professionals must be aware of the causes and risks of MARSI and provide proper care to prevent and treat those injuries effectively. We point out that early recognition is critical to ensure appropriate intervention and avoid loss of skin integrity and harm to the patient^(2,11,17,19)^.

It is necessary to highlight that each individual’s particularities intensify in the intensive care unit setting: the critically ill patient is more likely to be exposed to medical adhesives because the treatment requires the application of monitoring electrodes, vascular access fixation, and drain fixation, respiratory devices, and dressings. Moreover, patients are exposed to anticoagulants, vesicant medications, corticoids, mechanical ventilation, and tissue hypoxia^(1,7,18)^.

Considerations about the etiology of skin injuries are relevant to planning and developing strategies to prevent MARSI. Despite the data available in the literature, the professionals are still unfamiliar with the subject, which may be due to the lack of validated care protocols regarding MARSI.

### Study limitations

We excluded the terms associated with DAI and friction injury during the research protocol development because they were confounding injuries for MARSI. However, some MARSI can be reported in those studies.

### Contributions to the field of Nursing

This review contributes to translating scientific evidence into necessary actions in clinical practice. The results presented can support the development of care protocols and guide the actions of nursing professionals, aiming to improve the quality of health care and the safety of patients in critical care who routinely use medical patches. They also reinforce the need to publish studies with a strong level of evidence regarding nursing care to prevent MARSI in the adult intensive care setting.

## CONCLUSIONS

During the process of building this scoping review, the lack of studies addressing specifically MARSI preventive measures in the adult intensive care setting was evident. Mostly, there are no recommendations with a high level of evidence; and there is a lack of clinical studies on preventive strategies implemented in intensive care and their respective results. We point out that only one national study was identified, showing that the subject is still little published in Brazil.

The main strategies described in the studies correspond to skin assessment, identifying patients at risk, selecting the adequate adhesive product, the adequate technique for application and removal of the medical patch, and teaching professionals and patients.

Taking care of the skin of critically ill patients using medical adhesives is challenging. To do so, the professional must understand the importance of maintaining skin integrity and identify the characteristics of MARSI and the main strategies to prevent those injuries.
